# Supplementary material for: Traffic-Related Air Pollution and Breast Cancer Risk: A Systematic Review and Meta-Analysis of Observational Studies
Source: Cancers (Basel). 2023 Feb 1;15(3):927. doi: 10.3390/cancers15030927 (PMC9913524; doi:10.3390/cancers15030927)
Supplement: Supplementary file 1 [file cancers-15-00927-s001.zip › cancers-2148816-supplementary.pdf]

Supplementary materials

Material S1: PRISMA checklist

Section/topic	#	Checklist item	Reported on page #
**TITLE**			
Title	1	Identify the report as a systematic review, meta-analysis, or both.	1
**ABSTRACT**			
Structured summary	2	Provide a structured summary including, as applicable: background; objectives; data sources; study eligibility criteria, participants, and interventions; study appraisal and synthesis methods; results; limitations; conclusions and implications of key findings; systematic review registration number.	1
**INTRODUCTION**			
Rationale	3	Describe the rationale for the review in the context of what is already known.	2
Objectives	4	Provide an explicit statement of questions being addressed with reference to participants, interventions, comparisons, outcomes, and study design (PICOS).	2
**METHODS**			
Protocol and registration	5	Indicate if a review protocol exists, if and where it can be accessed (e.g., Web address), and, if available, provide registration information including registration number.	2
Eligibility criteria	6	Specify study characteristics (e.g., PICOS, length of follow-up) and report characteristics (e.g., years considered, language, publication status) used as criteria for eligibility, giving rationale.	3
Information sources	7	Describe all information sources (e.g., databases with dates of coverage, contact with study authors to identify additional studies) in the search and date last searched.	3
Search	8	Present full electronic search strategy for at least one database, including any limits used, such that it could be repeated.	3 and Materials S2 and S3
Study selection	9	State the process for selecting studies (i.e., screening, eligibility, included in systematic review, and, if applicable, included in the meta-analysis).	3
Data collection process	10	Describe method of data extraction from reports (e.g., piloted forms, independently, in duplicate) and any processes for obtaining and confirming data from investigators.	3
Data items	11	List and define all variables for which data were sought (e.g., PICOS, funding sources) and any assumptions and simplifications made.	3
Risk of bias in individual studies	12	Describe methods used for assessing risk of bias of individual studies (including specification of whether this was done at the study or outcome level), and how this information is to be used in any data synthesis.	4
Summary measures	13	State the principal summary measures (e.g., risk ratio, difference in means).	4
Synthesis of results	14	Describe the methods of handling data and combining results of studies, if done, including measures of consistency (e.g., I^2) for each	4

		meta-analysis.	
Risk of bias across studies	15	Specify any assessment of risk of bias that may affect the cumulative evidence (e.g., publication bias, selective reporting within studies).	4
Additional analyses	16	Describe methods of additional analyses (e.g., sensitivity or subgroup analyses, meta-regression), if done, indicating which were pre-specified.	4
**RESULTS**			
Study selection	17	Give numbers of studies screened, assessed for eligibility, and included in the review, with reasons for exclusions at each stage, ideally with a flow diagram.	5, Figure 1
Study characteristics	18	For each study, present characteristics for which data were extracted (e.g., study size, PICOS, follow-up period) and provide the citations.	6, 7, 9 Table 1, Figure 2
Risk of bias within studies	19	Present data on risk of bias of each study and, if available, any outcome level assessment (see item 12).	21
Results of individual studies	20	For all outcomes considered (benefits or harms), present, for each study: (a) simple summary data for each intervention group (b) effect estimates and confidence intervals, ideally with a forest plot.	21-23, Table 1, Figures 3 et 4
Synthesis of results	21	Present results of each meta-analysis done, including confidence intervals and measures of consistency.	21-23, Figures 3 et 4
Risk of bias across studies	22	Present results of any assessment of risk of bias across studies (see Item 15).	21
Additional analysis	23	Give results of additional analyses, if done (e.g., sensitivity or subgroup analyses, meta-regression [see Item 16]).	21, 22, 23, Table 2 and Tables S1 and S2 and Figure S1
**DISCUSSION**			
Summary of evidence	24	Summarize the main findings including the strength of evidence for each main outcome; consider their relevance to key groups (e.g., healthcare providers, users, and policy makers).	23
Limitations	25	Discuss limitations at study and outcome level (e.g., risk of bias), and at review-level (e.g., incomplete retrieval of identified research, reporting bias).	24,25, 26
Conclusions	26	Provide a general interpretation of the results in the context of other evidence, and implications for future research.	25, 26
**FUNDING**			
Funding	27	Describe sources of funding for the systematic review and other support (e.g., supply of data); role of funders for the systematic review.	26

From: Moher D, Liberati A, Tetzlaff J, Altman DG, The PRISMA Group (2009). Preferred Reporting Items for Systematic Reviews and Meta-Analyses: The PRISMA Statement. PLoS Med 6(7): e1000097. doi:10.1371/journal.pmed1000097 For more information, visit: **www.prisma-statement.org**.

Material S2: Research algorithm for the systematic review of the literature on breast cancer risk and exposure to traffic-related pollution using Medline/PubMed

Filter: English, French, German, Italian, Human
Equation: #1 OR #2 OR #3 AND #4 NOT #5 AND #6

Last search: June 2022

#1"Air Pollution"[Mesh: noexp] OR "Air Pollution, Indoor"[Mesh] OR "Air Pollutants"[Mesh: noexp] OR Air Pollut* [Tiab] OR Ambient Air[Tiab] OR Airborne Pollut* [Tiab] OR Air Toxic*[Tiab] OR Air quality[Tiab]
OR "Environmental Exposure"[Mesh:noexp]
OR ("Inhalation Exposure"[Mesh] OR Inhalation [Tiab] OR Inhale [Tiab] OR Inhaled [Tiab])

#2 "Vehicle Emissions"[Mesh] OR " Motor Vehicles" [Mesh] OR Traffic Pollut*[Tiab] OR Emissions [Tiab] OR Exhaust [Tiab] OR Exhausts [Tiab] OR Fume [Tiab] OR Fumes [Tiab]
OR ((Vehicle [Tiab] OR Vehicles [Tiab] OR Vehicular [Tiab] OR Auto [Tiab] OR Automobile [Tiab] OR Bus [Tiab] OR Buses [Tiab] OR Car [Tiab] OR Cars [Tiab] OR Truck [Tiab] OR Trucks [Tiab] OR Engine [Tiab] OR Transport [Tiab] OR Transportation [Tiab]) AND (Emissions [Tiab] OR Exhaust [Tiab] OR Fume [Tiab] OR Fumes [Tiab]))
OR (Traffic [Tiab] NOT (Safety [Tiab] OR Accident* [Tiab] OR Injur* [Tiab] OR Collision* [Tiab] OR Crash*[Tiab]))
OR ((Proximity [Tiab] OR Near [Tiab]) AND (Road [Tiab] OR Roadways [Tiab] OR Highway [Tiab] OR Highways [Tiab] OR Freeway [Tiab] OR Freeways [Tiab] OR Motorway [Tiab] OR Motorways [Tiab]))

#3
"Particulate Matter"[Mesh] OR Particulate Matter [Tiab] OR PM2.5 [Tiab] OR "PM(2.5)" [Tiab] OR PM10 [Tiab] OR "PM(10)" [Tiab] OR "Soot"[Mesh] OR Soot [Tiab] OR "black carbon" [Tiab]
OR "Benzo(a)pyrene"[Mesh] OR Benzene [Mesh] OR "Benzopyrene" [Tiab] OR "Benzo(a)pyrene"[Tiab] OR "3, 4-Benzopyrene" [Tiab] OR Benzene [Tiab]
OR "Nitrogen Dioxide"[Mesh] OR Nitrogen Dioxide* [Tiab] OR "NO(x)" [Tiab] OR NOx [Tiab] OR NO2 [Tiab] OR Nitrogen Oxide*[Tiab] OR Nitric Oxide [Tiab]
OR "Carbon Monoxide"[Mesh] OR "Carbon Monoxide"[Tiab]
OR "Volatile Organic Compounds"[Mesh] OR "Volatile Organic Compounds"[Tiab]

#4 "Breast Neoplasms"[Mesh] OR Breast cancer [Tiab] OR (Cancer [Tiab] AND Breast [Tiab])

#5 Treatment[Tiab] OR Therapy[Tiab] OR Radiotherapy[Tiab] OR Chemotherapy[Tiab] OR Immunotherapy[Tiab] OR Tomography[Ti] OR Mammograph*[ti] OR "Mammography"[Mesh] OR Imaging[Ti] OR"Sentinel Lymph Node Biopsy"[Mesh] OR Mastectomy[Ti] OR Nanoparticle[Ti] OR "Cell Line"[Mesh] OR Cell[Tiab] RNA[Ti] OR Management[Ti] OR Diagnosis[Ti] OR Prognosis[Mesh] OR Scintigraph*[Ti]

#6 "Clinical Trials as Topic"[Mesh] OR "Case-Control Studies"[Mesh] OR "Cohort Studies"[Mesh] OR "Cross-Sectional Studies"[Mesh] OR "Multicenter Studies as Topic"[Mesh] OR "Retrospective Studies"[Mesh] OR "Epidemiology"[Mesh] OR "Epidemiology" [Subheading]

Material S3: Research algorithm for the systematic review of the literature on breast cancer risk and exposure to traffic-related pollution using Web of science

Filter: English, Article
Equation: (((#1 OR #2 OR #3) AND #4) NOT #5) AND #6

Last search: June 2022

#1
TS=("Air Pollution" OR "Air Pollutants" OR "Environmental Exposure" OR "Inhalation Exposure") OR TI=(Air Pollut* OR Ambient Air OR Airborne Pollut* OR Air Toxic* OR Air quality) OR
AB=(Air Pollut* OR Ambient Air OR Airborne Pollut* OR Air Toxic* OR Air quality) OR AK=(Air Pollut* OR Ambient Air OR Airborne Pollut* OR Air Toxic* OR Air quality)

#2
TS=("Vehicle Emissions" OR " Motor Vehicles") OR TI=(Traffic Pollut OR Emissions OR Exhaust OR Exhausts OR Fume OR Fumes) OR TI=((Vehic* OR Automobile OR Bus OR Buses OR Car OR Cars OR Truck OR Trucks OR Engine OR Transport OR Transportation) AND (Emissions OR Exhaust OR Fume OR Fumes)) OR TI=(Traffic NOT (Safety OR Accident* OR Injur* OR Collision* OR Crash*)) OR TI=((Proximity OR "Near") AND (Road OR Roadways OR Highway OR Highways OR Freeway OR Freeways OR Motorway OR Motorways))

#3
TS=("Particulate Matter" OR "$PM_{2.5}$" OR "PM(2.5)" OR PM10 OR "PM(10)" OR Soot OR "black carbon" OR "Benzo(a)pyrene" OR Benzene OR "Nitrogen Dioxide" OR "Carbon Monoxide" OR "Volatile Organic Compounds") OR
TI=("Particulate Matter" OR "$PM_{2.5}$" OR "PM(2.5)" OR PM10 OR "PM(10)" OR Soot OR "black carbon" OR "Benzo(a)pyrene" OR "Benzopyrene" OR "Benzo(a)pyrene" OR Benzene OR "3, 4-Benzopyrene" OR "Nitrogen Dioxide" OR "NO(x)" OR NOx OR NO2 OR "Nitrogen Oxide" OR "Nitric Oxide" OR "Carbon Monoxide" OR "Volatile Organic Compounds")

#4
TS=("Breast Neoplasms") OR
TI=("Breast cancer" OR (Cancer AND Breast))

#5
TI=(Treatment OR Therapy OR Radiotherapy OR Chemotherapy OR Immunotherapy OR Tomography OR Mammograph* OR "Mammography" OR Imaging OR "Sentinel Lymph Node Biopsy" OR Mastectomy OR Nanoparticle OR "Cell Line" OR RNA OR Management OR Diagnosis OR Prognosis OR Scintigraph*)

#6
TS=("Clinical Trials as Topic" OR "Case-Control Studies" OR "Cohort Studies" OR "Cross-Sectional Studies" OR "Multicenter Studies as Topic" OR "Retrospective Studies" OR "Epidemiology")

Table S1: Summary relative risks (RR) and 95% confidence interval (CI) of breast cancer for an increase of 10 μg/m^3 of NO_2, overall and excluding one study at a time.

	N studies	RR (95% CI)	I^2 (%)	*p* for heterogeneity	ID of included articles [a]
Overall meta-estimate	13	1.0151 (1.0026, 1.0279)	16.9	0.27	1-6, 8-14
Excluding Amadou et al. 2022	12	1.0132 (1.0005, 1.0260)	15.0	0.30	1-6, 8-13
Excluding Lemarchand et al. 2021	12	1.0132 (1.0020, 1.0245)	10.6	0.34	1-6, 8-11, 13, 14
Excluding White et al. 2021	12	1.0180 (1.0041, 1.0321)	19.3	0.25	1-6, 8-11, 13, 14
Excluding Cheng et al. 2020	12	1.0166 (1.0022, 1.0312)	23.8	0.21	1-6, 8-14
Excluding Bai et al. 2019	12	1.0202 (1.0033, 1.0373)	15.5	0.29	1-6,8,9,11-14
Excluding Goldberg et al. 2019	12	1.0186 (1.0039, 1.0335)	20.7	0.24	1-6,8,10-14
Excluding White et al. 2019	12	1.0166 (1.0024, 1.0309)	20.7	0.26	1-6, 9-14
Excluding Datzmann et al. 2018	12	1.0090 (1.0009, 1.0172)	0.0	0.79	1-5, 8-14
Excluding Andersen et al. 2017b	12	1.0157 (1.0015, 1.0301)	23.0	0.22	1-4, 6, 8-14
Excluding Andersen et al. 2017a	12	1.0163 (1.0026, 1.0302)	23.5	0.21	1-3, 5, 6, 8-14
Excluding Goldberg et al. 2017	12	1.0156 (1.0023, 1.0292)	22.7	0.22	1, 2, 4-6, 8-14
Excluding Hystad et al. 2015	12	1.0152 (1.0019, 1.0287)	21.6	0.23	1, 3-6, 8-14
Excluding Crouse et al. 2010	12	1.0146 (1.0024, 1.0270)	8.6	0.36	2-6, 8-13, 14

[a] Study IDs are listed in Table 1

Table S2: Summary relative risks (RR) and 95% confidence interval (CI) of breast cancer for an increase of 10 μg/m³ of NO_2, cumulating studies by order of publication

ID of included articles [a]	N studies	RR (95% CI)	I^2 (%)	*p* for heterogeneity
1,2,3	3	1.0536 (0.9664, 1.1487)	0.0	0.59
1-4	4	1.0268 (0.9655, 1.0918)	0.0	0.62
1-5	5	1.0223 (0.9864, 1.0595)	0.0	0.77
1-6	6	1.0421 (1.0142, 1.0708)	0.0	0.49
1-6, 8	7	1.0345 (1.0101, 1.0595)	0.0	0.46
1-6, 8,9	8	1.0291 (1.0077, 1.0509)	0.0	0.49
1-6, 8-10	9	1.0203 (1.0012, 1.0496)	9.4	0.36
1-6, 8-11	10	1.0134 (1.0011, 1.0260)	10.8	0.34
1-6, 8-12	11	1.0111 (1.0004, 1.0219)	7.2	0.37
1-6, 8-13	12	1.0132 (1.0005, 1.0260)	15.0	0.30
1-6, 8-14	13	1.0151 (1.0026, 1.0279)	16.9	0.27

[a] Study IDs are listed in Table 1

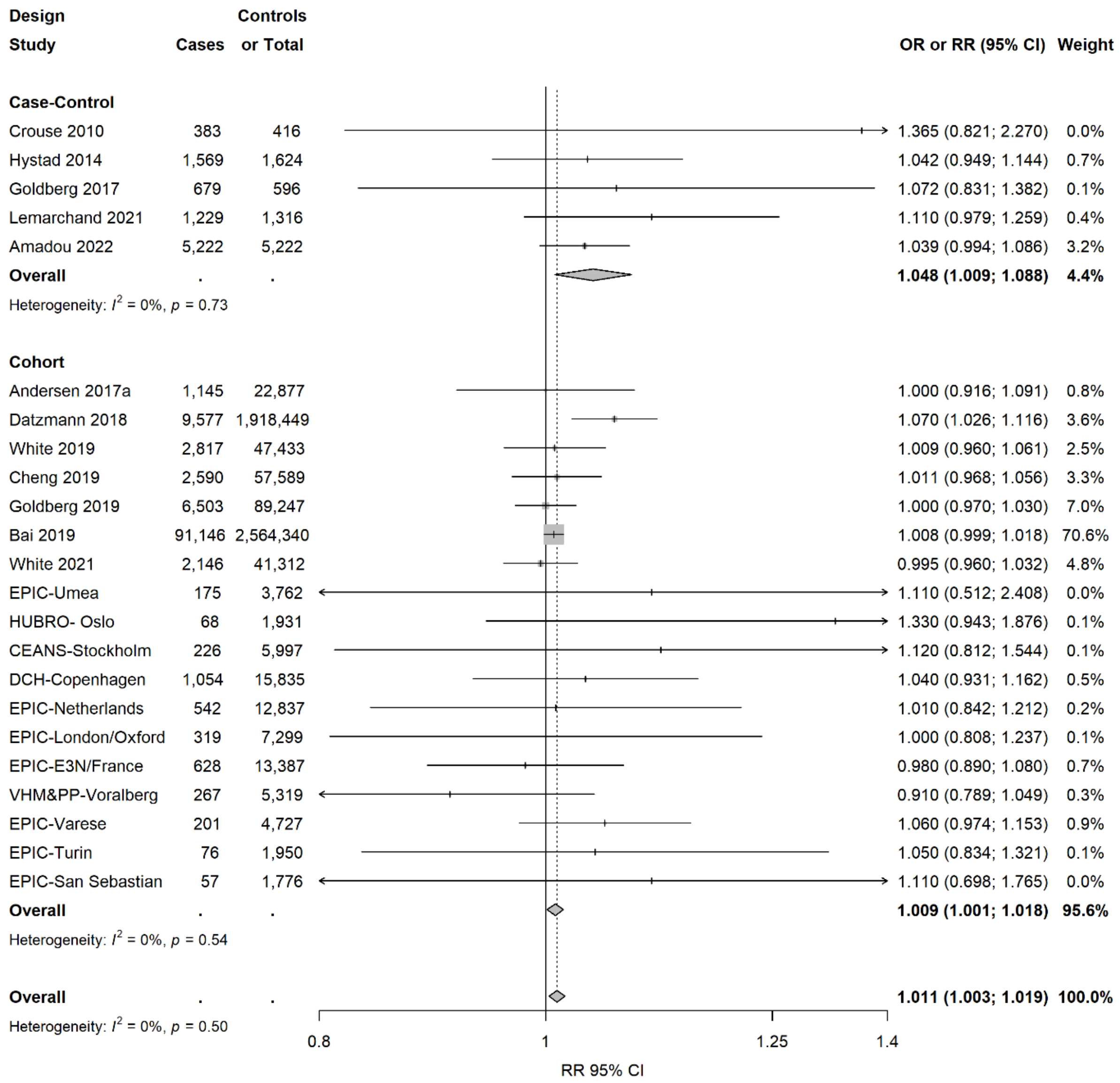

Figure S1: Study-specific and summary RRs and 95% confidence intervals for the association between invasive breast cancer and exposure to an increase of 10 μg/m³ in NO_2 including studies of the ESCAPE project individually.
